# “Let’s Get Out of Here!”: Cognitive Motivation and Maximizing Help Teams Solving an Escape Room

**DOI:** 10.3389/fpsyg.2020.02196

**Published:** 2020-08-31

**Authors:** Vidar Schei, Therese Egeland, Elisabeth Andvik

**Affiliations:** Department of Strategy and Management, NHH Norwegian School of Economics, Bergen, Norway

**Keywords:** team, cognitive motivation/need for cognition, maximizing, cooperation, escape room

## Abstract

Contemporary teams often face complex problem-solving tasks. We theorized that two individual differences previously neglected in team research (cognitive motivation and maximizing) would be helpful for teams facing such situations. We tested this assertion on 81 teams participating in an escape-room simulation in which teams were locked into a pre-arranged room and had to solve various complex problems to escape the room as quickly as possible. The findings show that the average of the team members’ cognitive motivation had a positive direct relation to team performance, while maximizing had a positive indirect relation to team performance via cooperation.

## Introduction

Organizations today face technological changes, globalization, and competition, all of which have led to tasks that are too complex for an individual to solve alone (Burke et al., 2006). In response, organizations structure work around teams in order to draw on a broader range of competencies, capacities, and experiences (Santos et al., 2015). Hence, organizational success comes from the ability of a team to collaborate effectively and to solve complex and unstructured problems (DeChurch and Mesmer-Magnus, 2010). Complex problem solving in teams requires both cognitive and social skills in that the team members must collaborate on defining the problem (social skills) and combine their individual resources (personality, cognitive skills, etc.) (Graesser et al., 2018). However, available empirical findings lack a comprehensive understanding of which individual compositional variables are important in this regard and how they are related to team process variables such as collaboration (Graesser et al., 2018).

Thus, the present study aims to examine the roles of individual differences in teams that face complex problems and how team members collaborate to solve these problems. We suggest two individual differences that are rather novel to team research – cognitive motivation and maximizing – as promising candidates to help teams solve complex problems. *Cognitive motivation*, the willingness to engage in arduous, analytical thinking (Chatterjee et al., 2000), can stimulate team members to use their cognitive capacity to the fullest and discuss the task carefully. Similarly, *maximizing*, the tendency to look for the best choice (Schwartz et al., 2002), can push team members to persevere even when conditions become challenging and frustrating, as is likely to be the case in complex circumstances. These individual differences may affect how team members cooperate, the latter being an important process variable with regard to team performance (e.g., Stewart and Barrick, 2000). Hence, we were interested in investigating whether cooperation would mediate the relationships between cognitive motivation and maximizing and team performance.

We examined these relationships in a sample of 81 teams participating in an escape-room exercise. The teams were locked into a room, especially designed for the occasion, filled with puzzles that had to be discovered, interpreted, and combined to open a series of locks in order to escape from the room as quickly as possible. Thus, the goal was clear, but structuring the process and organizing the team to solve the task presented highly complex challenges.

### Complex Problem Solving in Teams

Complex problem solving concerns reducing the barrier between start and end goal with the help of cognitive activities and behavior (Funke, 2012). Teams that are highly interdependent require a higher degree of interaction compared to teams with lower levels of interdependence (Wageman, 1995), and this also requires various cognitive and social skills (Hagemann and Kluge, 2017).

Hagemann and Kluge (2017) presented a teamwork process model that applies to complex problem-solving teams, where they suggest various teamwork competencies and teamwork processes that affect team performance in complex environments. In short, the model suggests that team members interact interdependently and convert inputs to outcomes through cognitive verbal and behavioral activities. They empirically tested the model and found that collective orientation of team members affects team performance mediated by coordination. However, they stressed that the model should be tested in other settings and also that other variables tied to team composition and processes are viable. The model builds on Ilgen et al. (2005) framework of I (input)–P (process)–O (output) with cyclical episodes, highlighting the importance of coordination, cooperation, and communication for effective action processes related to problem solving. We build on this model and suggest that input variables related to team members’ need for cognition and maximizing could potentially be important for process variables like coordination, cooperation, and communication.

In the following, we address the literature on cognitive motivation and maximization, respectively, to develop hypotheses on how these individual differences relate to cooperation and performance in complex problem-solving tasks. Our interest is to examine these relationships at the team level, i.e., how cognitive motivation and maximization in teams are associated with team cooperation and team performance. However, as the research on these individual differences at the team level is relatively scarce, we also draw on research at the individual level to develop our hypotheses.

### Cognitive Motivation

Cognitive motivation, or need for cognition, is a relatively stable individual difference (Bruinsma and Crutzen, 2018) in the tendency to engage in and enjoy effortful cognitive activities (Chatterjee et al., 2000). Thus, cognitive motivation concerns the willingness to invest in information-processing endeavors rather than cognitive ability and is therefore only modestly correlated with cognitive intelligence (Cacioppo et al., 1996).

At the individual level, previous research has consistently found cognitive motivation to be helpful in complex problem solving (Cacioppo et al., 1996). For instance, individuals with high cognitive motivation actively search for information (Verplanken et al., 1992), while individuals with low cognitive motivation are more susceptible to cognitive heuristics (Cacioppo et al., 1996). Moreover, individuals with high cognitive motivation are more creative (Hahn and Lee, 2017) and curious (Olson et al., 1984) and therefore are effective problem solvers in unstructured tasks (Nair and Ramnarayan, 2000) compared to those with low cognitive motivation, who are more easily stressed by complex cognitive tasks (Gülgöz, 2001). Individuals scoring high on cognitive motivation are also found to perform better on academical tests because of their effective information processing (Sadowski and Gülgöz, 1996), to score better on retention tests (Stenlund et al., 2017), and to process information with greater depth and breadth (Levin et al., 2000). Thus, the findings at the individual level show that cognitive motivation is a very useful trait in complex problem-solving situations in which identifying cues, seeking information, and thriving under challenging cognitive conditions are crucial to success.

At the team level, our knowledge of the effects of cognitive motivation is quite limited. Emerging evidence nevertheless indicates that cognitive motivation may be helpful at aggregated levels. For example, Smith et al. (2001) found student teams with high cognitive motivation to be less prone to social loafing on a cognitively engaging task than teams with low cognitive motivation. Similarly, Curseu (2011) found that team members with high cognitive motivation seek more advice in task-related issues. Also, student teams with higher levels of cognitive motivation have higher-quality discussions (Curseu et al., 2018) and are reported to have higher teamwork quality (Curseu and Pluut, 2013). These findings indicate that teams with high cognitive motivation may be more likely to exchange information and cooperate. This is further supported by findings showing that those high in cognitive motivation are less susceptible to stereotypical views of others (e.g., Carter et al., 2006). Teams with higher average scores on cognitive motivation are also found to be better at generating alternatives in brainstorming tasks than are teams with lower cognitive motivation (Scudder et al., 1994). Furthermore, Kearney et al. (2009) reported a positive bivariate correlation between cognitive motivation and performance in teams involved in knowledge-based tasks, such as the development of new products and services. Similarly, in work teams from Chinese organizations, high cognitive motivation in the team was positively related to leader-rated team performance (Chen et al., 2012). Finally, in research linking cognitive motivation to performance via team processes, the cognitive motivation of sellers was found to enhance the providing of information in integrative negotiation processes, which in turn improved joint outcomes (Schei et al., 2006).

Thus, we suggest that, in complex problem-solving situations, team members’ cognitive motivation is directly positively related to team performance and indirectly related via cooperation (Hypothesis 1).

### Maximizing

Maximizing is an individual difference in the desire to achieve the best outcome when making decisions (Schwartz et al., 2002). Maximizing is sometimes portrayed as the opposite of satisficing, i.e., the tendency to settle for an option that is good enough. Maximizing is related to, but different from, perfectionism and has been found to be moderately correlated with various measures of perfectionism (Chang et al., 2011).

At the individual level, research on maximizing mostly addresses decision making by consumers. For instance, Schwartz et al. (2002) found that high maximizers in general exhibited more thorough decision processes than low maximizers. That is, high maximizers were found to engage in more product comparisons and more counterfactual thinking regarding purchases. Others have found maximizers to be more willing to sacrifice resources such as time in order to acquire more alternatives to choose from Dar-Nimrod et al. (2009). Maximizers were also found to have higher numerical skills than others (Misuraca et al., 2015), which may be beneficial when solving complex problems that require calculations. Moreover, high maximizers have been shown to achieve better academic performance in business school (Stohs, 2016) and have also been tied to job satisfaction and performance (e.g., Iyengar et al., 2006; Giacopelli et al., 2013).

At the team level, no studies seem to have examined maximizing in a group setting directly. However, the findings from consumer-behavior research indicate that high-maximizing teams will search for a multitude of alternatives. We believe that in complex problem-solving situations in particular, where searching for clues and solutions is likely to be important, teams with high-maximizing members should have an advantage. High-maximizing teams are more likely to avoid the “stopping rule” (Cheek and Schwartz, 2016) and to search continually for better options. The tendency to persist in looking for various alternatives is particularly important in the current setting in order to identify and combine information that is available in the environment (i.e., the escape room). Thus, teams of high maximizers are likely to solve more problems than teams with non-maximizers. Moreover, this tendency to maximize may also motivate team members to cooperate well in order to excel in their problem-solving efforts and not be satisfied with solving only parts of the problems they encounter. Cooperating with others on the team appears to be particularly attractive for maximizers, as this gives them better opportunities to map out as many alternatives as possible.

Thus, we suggest that, in complex problem-solving situations, team members’ maximizing is directly positively related to team performance and indirectly related via cooperation (Hypothesis 2).

### Aggregation

Cognitive motivation and maximizing are both individual-level constructs. By moving from the individual level to the group level, we encounter several challenges to exploring whether and how individual-level variables influence team performance. Research suggests that the appropriateness of an aggregation method depends on the type of task and the type of trait (e.g., Prewett et al., 2009).

The *task approach* relies on typologies such as Steiner’s (1972) group model, which distinguishes between additive, disjunctive, and conjunctive tasks. In the present study, we argue that the task used is primarily additive. Additive tasks are those where the contributions of the individual members are combined into the final team product, and performance should therefore be best predicted by the mean of the individual abilities in question. The teams in our study faced a complex task that was almost impossible for one or a few members to solve alone. The *trait approach* asserts that the fit between an individual ability and the team may be either supplementary or complementary (Prewett et al., 2009). In the present study, we argue that the individual differences are primarily supplementary. A supplementary fit posits that to optimize performance, the team members should have similar levels of the trait. Thus, consistently high values on cognitive motivation and maximization within the teams in our study are likely to be better than high variation.

Consequently, we suggest that mean scores are an appropriate aggregation method in the current study. This approach is also consistent with recent meta-analytical findings on the team personality–team performance link (Prewett et al., 2009), as well as research by LePine et al. (2011) finding the mean level of personality characteristics to “consistently predict outcomes as well as, if not better than, the minimum, maximum, or variance operationalizations” (p. 326). However, to give a more refined picture of our findings, and as recommended in previous research on team ability (e.g., Barrick et al., 1998), we will explore different ways of aggregating cognitive motivation and maximizing in our supplemental analyses.

## Methods

This study is part of a larger dataset where we followed student teams in several activities for some months, gathering data for testing, exploratory, and pedagogical purposes. For this article, we examined the escape-room exercise with two independent variables (cognitive motivation and maximizing), a generic process variable (cooperation), and objective team performance. The study was approved by the Norwegian Centre for Research Data.

### Participants and Procedure

The participants were 330 undergraduate students (M_age_ = 21.8; 39% female) in an organizational behavior course at a leading Norwegian business school. Early in the semester, the students chose 1 of 11 classes that fit their schedule. Within the classes, participants were randomly assigned to 81 teams, comprising 3 three-member teams, 69 four-member teams, and 9 five-member teams. The escape-room simulation took place about 1 month after the teams were formed and answered online questionnaires measuring cognitive motivation and maximizing. The teams participated in at least one exercise before the escape-room simulation and thus had some prior history of working together. Following the exercise, the participants answered a short questionnaire that included a cooperation measure.

### Task

Escape rooms have become quite popular since the first rooms appeared in 2007, there being almost 1,800 registered rooms worldwide by 2015 (French and Shaw, 2015), increasing to probably more than 10,000 rooms by the end of 2018 (The Economist, 2019). An escape room is an organized game that takes place in a closed room. Typically, a team of two to six players is locked into the room and works together to solve various puzzles. The various tasks demand the use of logical and analytical skills as well as creativity and team collaboration. A typical task involves looking for patterns, for example, determining what the correct time should be on a fourth clock by looking at the pattern from three previous clocks. Another typical task might involve using something in the room in an unusual way. If the team solves a puzzle, they receive a code or key that fits a padlock in the constructed room. The goal is to escape the room as quickly as possible and before a 60 min deadline. We engaged a professional firm, www.escapebryggen.no, to develop a room for this study, having the teams face a complex problem-solving task that was almost impossible for one or a few members to solve alone (i.e., cooperation was required). An escape room “enables novel insights into team processes and performance through interaction of escape room elements with teamwork and problem solving” (Cohen et al., 2020, p. 14). Such rooms are reminiscent of tasks that are used in assessment centers in that people are put in groups to solve complex tasks in more or less realistic situations and are observed while collaborating (Dimotakis et al., 2017). Assessment centers are popular for the recruitment, selection, management, and leadership of development programs because of their strong criterion-related validity evidence and their apparent objectivity compared to other alternatives (Meriac et al., 2008).

### Variables

We measured *team performance* as the number of locks the groups were able to open in the escape room during the 1 h session. The number of locks opened was assessed by looking at videotapes from cameras inside the room. The groups had to open 14 locks to escape. Only one group was able to escape before the deadline.

The survey measures ranged from 1 (disagree) to 5 (agree). We measured *cognitive motivation* with the short version of the Need for Cognition Scale (Cacioppo et al., 1984). The scale consists of 18 items, e.g., “I prefer complex rather than simple problems.” We measured *maximizing* with the Maximization Scale (Schwartz et al., 2002). The scale consists of 13 items, e.g., “No matter what I do, I have the highest standards for myself.” We measured *cooperation* with three items: “Overall, I think we cooperated well during the exercise,” “I am disappointed with the way we worked on this exercise” (reverse-coded), and “I am pleased with how the group functioned in this exercise.” We included team start as a dichotomous *control variable* in all analyses, as, 1 month before the escape-room exercise, half the groups performed a short team-building exercise, while the other half wrote a team charter. Team start did not correlate significantly with any of the variables in this study, and the results remained the same whether it was included or not.

### Analyses

We measured cognitive motivation, maximizing, and cooperation at the individual level and aggregated them to the team level by averaging the team members’ scores in our primary analyses. The mean interrater agreement (James et al., 1984) for cooperation was 0.70, and the intraclass correlations (ICC1 and ICC2) were 0.47 and 0.79, respectively. These scores are all considered to be strong (LeBreton and Senter, 2008; Biemann et al., 2012). We analyzed the data in SPSS by regression analyses to test for direct effects and by Hayes’s process macro (3.1) to test for indirect effects (Hayes, 2018).

## Results

The descriptive statistics, correlations, and coefficient alphas are shown in Table 1. Team performance had a mean score of 7.88; thus, teams on average were able to unlock almost 8 of the 14 keys in the room. Cognitive motivation and cooperation were significantly positively correlated with team performance. Maximizing was also positively, but non-significantly, related to team performance.

**TABLE 1:** Descriptive statistics, correlations, and coefficient alphas.

Variable	*M*	*SD*	1	2	3	4
1. Team performance	7.88	2.65				
2. Cognitive motivation	3.63	0.28	0.24*	0.86		
3. Maximizing	3.01	0.30	0.13	–0.03	0.64	
4. Cooperation	4.08	0.46	0.42***	0.12	0.29*	0.80

N = 81 teams for performance, cognitive motivation, and maximizing. N = 75 for cooperation, as six teams did not answer the post-simulation questionnaire. Coefficient alphas are shown in the diagonal. *p < 0.05, ***p < 0.001.

### Direct Effects

The regression model, including cognitive motivation and maximizing as independent variables and team performance as the dependent variable, was statistically significant [*ΔR^2^* = 0.08, *F*_(__2,__77__)_ = 3.26, *p* = 0.044] as shown in Table 2 (Model 1). Cognitive motivation was positively and statistically significantly related to team performance (*β* = 0.25, *p* = 0.026), while maximizing was not (*β* = 0.13, *p* = 0.227). Thus, the direct effect in Hypothesis 1 (cognitive motivation) was supported, but not the direct effect in Hypothesis 2 (maximizing).

**TABLE 2:** Results of regression analyses.

Variable	Model 1	Model 2
Cognitive motivation	0.25*	0.13
Maximizing	0.13	0.03
Cooperation		0.40**
*ΔR^2^*	0.08	0.14
*F* for *ΔR*^2^	3.26*	13.08**

N = 81 teams for Model 1. N = 75 for Model 2, as six teams did not answer the post-simulation questionnaire. Both models are controlled for team-start intervention (entered in a first step not shown in the table). Standardized coefficients are shown. *p < 0.05, **p < 0.01.

### Indirect Effects

**FIGURE 1:**
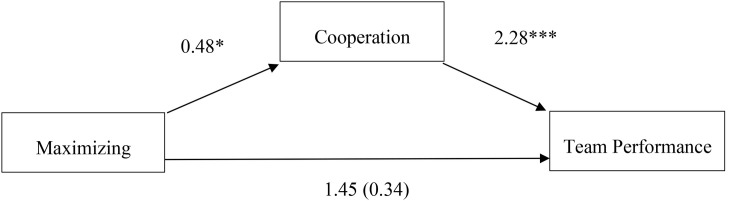
Indirect effects of maximizing on team performance via cooperation. Indirect effect: 95% CI [0.20, 2.30]. ^∗^*p* < 0.05, ^∗∗∗^*p* < 0.001.

The regression model, including cooperation, demonstrated that cooperation increased the explanatory power significantly (*F* for *ΔR*^2^ = 13.08, *p* = 0.001), as shown in Table 2 (Model 2). The bootstrap analysis with 5,000 samples for cognitive motivation and team performance via cooperation was not statistically significant [indirect effect: 95% confidence interval (−0.66, 1.75)]. The bootstrap analysis with 5,000 samples for maximizing and team performance via cooperation showed statistically significant effects [indirect effect: 95% confidence interval (0.20, 2.30)] and is shown in Figure 1. Thus, the indirect effect in Hypothesis 2 (maximizing) was supported, but not the indirect effect in Hypothesis 1 (cognitive motivation).

### Supplementary Analyses

We found no significant interaction effects of cognitive motivation and maximizing. We further examined the effects of cognitive motivation and maximizing, respectively, via alternative aggregations (minimum, maximum, and variance). For cognitive motivation, we found a statistically significant positive direct effect on team performance when aggregation was based on the lowest-scoring team member (*β* = 0.23, *p* = 0.036). For maximizing, we found a statistically significant positive indirect effect via cooperation when aggregation was based on the highest-scoring team member [indirect effect: 95% confidence interval (0.15, 1.71)].

## Discussion

This study aimed to examine how the individual differences of cognitive motivation and maximizing in teams facing complex problem-solving situations related to cooperation and team performance. We addressed this question by studying the performance of 81 teams locked into an escape room. Team cognitive motivation was positively related to team performance, while team maximizing was positively related to team performance via team cooperation.

### Contributions

The present research contributes to research on complex problem solving in teams by drawing attention to two individual differences that have been more or less neglected in previous research in these tasks. The positive effect of cognitive motivation corroborates the findings that have consistently been found in individual problem-solving situations. However, we were not able to show that the effect worked through cooperation, and future research will have to investigate why cognitive motivation works at the team level. Conversely, the positive effect of maximization worked only indirectly through cooperation. Hence, teams of maximizers should be aware that their joint potential is realized only when they collaborate with each other. Future research may examine the interplay between cognitive motivation, maximization, and other process variables. Interestingly, our supplemental analyses demonstrated that the cognitive motivation of the lowest-scoring member of a team was positively related to team performance. Thus, to solve complex problem-solving tasks, every team member may need to contribute with relatively high cognitive motivation. On the contrary, one member high in maximizing is sufficient to positively affect performance via cooperation. This may possibly be due to the high maximizer’s ability to generate alternatives, which the other team members may exploit in solving the problems. These findings extend previous research on complex problem solving in teams.

The present research also contributes by its rather novel methodological setup. Escape rooms are a well-established activity in a number of cities throughout the world and may constitute an interesting arena for conducting research on teams. Although escape rooms are rapidly growing in popularity in, for example, educational settings, “empirical research using escape rooms is limited” (Cohen et al., 2020, p. 14), despite the potential for designing rooms and tasks that are appropriate for a variety of more specific research questions. Importantly, escape rooms may resemble the complex and chaotic structure and the unclear procedure for addressing problems found in many of today’s teams. These rooms are also akin to the tasks found in the broader literature on assessment centers: tasks that have been shown to have high validity in, for example, recruitment and leadership development. These types of task are particularly relevant for teams where performance relies heavily on dimensions such as consideration/awareness of others, communication, drive, influencing others, organizing and planning, problem solving, and stress tolerance (Meriac et al., 2008).

### Limitations and Further Research

As is typically the case in this kind of research, it may be other variables, not measured, that explain team performance. Cognitive ability is an obvious candidate, but previous research has shown that IQ is only moderately correlated to variables such as cognitive motivation (Cacioppo et al., 1996). Furthermore, our findings may be restricted to the context of students in an escape room. Although our students in general seemed motivated and dedicated, further research should examine whether our findings hold outside the specific setting. Future studies might also include samples that could be expected to have greater variance in participants’ cognitive motivation and maximizing than was the case in our study. Another avenue for further investigation is to look at the more fine-grained effects of cognitive motivation and maximizing under different circumstances (e.g., different types of tasks) and under different goal and information structures (e.g., Wittenbaum et al., 2004) to better reveal the scope of the effects. Finally, future research may want to look more deeply into matters of aggregation to assess whether collective effects of these individual differences exist that are not accounted for by mere analyses of means, variance, and extreme scores (see, for example, the findings on group collective intelligence of Woolley et al., 2010).

## Conclusion

Contemporary teams increasingly face important challenges in complex problem-solving tasks. Thus, it is essential to determine the factors that may help teams to handle these situations effectively. Our study provides promising results on two individual differences that have been largely neglected in previous team research on complex problem solving. We hope that these findings inspire future researchers to elaborate on the potential power of individual differences in teams facing challenging tasks.

## Data Availability Statement

The datasets generated for this study are available on request to the corresponding author.

## Ethics Statement

The studies involving human participants were reviewed and approved by the NSD Norwegian Data Protection Services. The patients/participants provided their written informed consent to participate in this study.

## Author Contributions

VS, TE, and EA contributed to the idea and design of the study. VS did the statistical analysis and wrote the first draft of the manuscript. All authors contributed to manuscript revision, read and approved the submitted version.

## Conflict of Interest

The authors declare that the research was conducted in the absence of any commercial or financial relationships that could be construed as a potential conflict of interest.
